# Down regulation of *p-coumarate 3-hydroxylase* in petunia uniquely alters the profile of emitted floral volatiles

**DOI:** 10.1038/s41598-019-45183-2

**Published:** 2019-06-20

**Authors:** Joo Young Kim, Robert T. Swanson, Maria I. Alvarez, Timothy S. Johnson, Keun H. Cho, David G. Clark, Thomas A. Colquhoun

**Affiliations:** Environmental Horticulture Department, Plant Innovation Center, Institute of Food and Agricultural Sciences, University of Florida, Gainesville, FL 32611 USA

**Keywords:** Plant physiology, Metabolomics

## Abstract

*Petunia* × *hybrida* cv ‘Mitchell Diploid’ floral volatile benzenoid/phenylpropanoid (FVBP) biosynthesis ultimately produces floral volatiles derived sequentially from phenylalanine, cinnamic acid, and *p*-coumaric acid. In an attempt to better understand biochemical steps after *p*-coumaric acid production, we cloned and characterized three petunia transcripts with high similarity to *p-coumarate 3-hydroxylase* (*C3H*), *hydroxycinnamoyl-CoA:shikimate/quinate hydroxycinnamoyl transferase* (*HCT*), and *caffeoyl shikimate esterase* (*CSE*). Transcript accumulation of *PhC3H* and *PhHCT* was highest in flower limb tissue during open flower stages. *PhCSE* transcript accumulation was also highest in flower limb tissue, but it was detected earlier at initial flower opening with a bell-shaped distribution pattern. Down regulation of endogenous *PhC3H* transcript resulted in altered transcript accumulation of many other FVBP network transcripts, a reduction in floral volatiles, and the emission of a novel floral volatile. Down regulation of *PhHCT* transcript did not have as large of an effect on floral volatiles as was observed for *PhC3H* down regulation, but eugenol and isoeugenol emissions were significantly reduced on the downstream floral volatiles. Together these results indicate that *PhC3H* is involved in FVBP biosynthesis and the reduction of *PhC3H* transcript influences FVBP metabolism at the network level. Additional research is required to illustrate *PhHCT* and *PhCSE* functions of petunia.

## Introduction

Plants have ability to produce specialized metabolites classified as phenylpropanoids. Thousands of distinct chemical structures are present in this class of organic compounds, ultimately all derived from the aromatic amino acid phenylalanine^1,2^. Many of these compounds and their respective conjugates are crucial for various function in plants: e.g. lignins, suberins, tannins (biopolymers - structural support, integrity, and pathogen resistance); flavonoids, isoflavonoids (pigments, sunscreens, and biotic interactions), and benzenoids/phenylpropenes (pollinator attractants, florivore repellents, and antimicrobials). Levels of these phenylpropanoid compounds can fluctuate drastically in response to abiotic and biotic stimuli^1^.

The initial enzymatic sequence of the phenylpropanoid pathway consists of phenylalanine ammonia-lyase (PAL), cinnamate 4-hydroxylase (C4H), and 4-coumaric acid CoA ligase (4CL)^3^. PAL catalyzes the non-oxidative deamination of L-phenylalanine to cinnamic acid and ammonia^4–6^. Distinct PAL gene variants demonstrate differential transcript accumulation in tissue specific patterns, throughout development, and after specific stimuli. This suggests discrete and individual functions for each of the corresponding PAL isozymes^7–10^. PAL proteins are, for the most part, soluble and localized to the cytosol^11–15^. Functional PAL enzymes are usually identified as a homotetrameric protein aggregate^1,16,17^.

C4H is a cytochrome P450-dependent monooxygenase and consumes molecular oxygen and utilizes NADPH to catalyze the hydroxylation of cinnamic acid to produce *p*-coumaric acid^18^. Transcript accumulation of C4H is identified in most plant tissues, at some level, and can be regulated by numerous abiotic and biotic stimuli^5,19–22^. C4H protein is localized to the endoplasmic reticulum (ER) where it is anchored to the cytoplasmic surface of the membrane by an N-terminal leader/targeting peptide^11,12,23^, and functional C4H enzyme is usually identified in a homo/heterodimeric form, although some evidence exists for heterotrimeric interactions with other ER bound proteins^11,24^.

4CL catalyzes the reactions of cinnamic acid derivatives into the corresponding CoA thioesters including *p*-coumaroyl-CoA, caffeoyl-CoA, feruloyl-CoA, 5-hydroxyferuloyl-CoA, and sinapoyl-CoA^9,25^. Multiple 4CL genes detected in several species were regulated differentially according to the tissues, developmental stage, and environmental stress, which lead to specific biological processes such as monolignol biosynthesis and flavonoid production^9,26^. The substrate preference for each 4CL isoform has not been elucidated yet^25^, and 4CL protein is localized to the cytosol and re-localized through interactions with other proteins including C3H, HCT near to the ER^15,25^.

**Figure 1:**
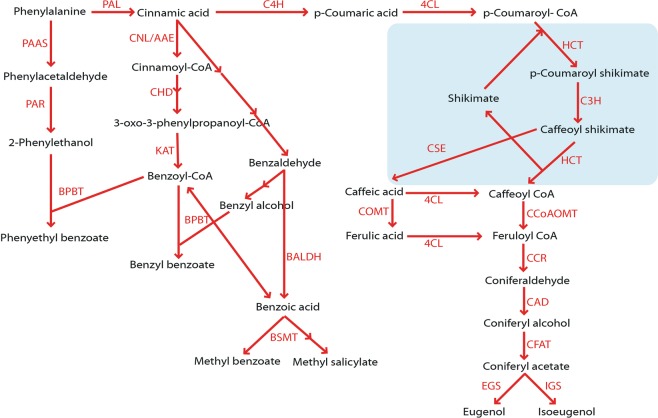
Proposed FBVP biosynthesis in *Petunia* ‘MD’. *BALDH*, benzaldehyde dehydrogenase; *BPBT*, benzoyl-CoA:benzylalcohol/2-phenylethanol benzoyltransferase; *BSMT*, benzoic acid/salicylic acid carboxyl methyltransferase; *4CL*, 4-coumaroyl-CoA ligase; *C3H*, *p*-coumarate 3-hydroxylase; *C4H*, cinnamate 4-hydroxylase; *CAD*, cinnamyl alcohol dehydrogenase; *CCoAOMT*, caffeoyl-CoA 3-O-methyltransferase; *CCR*, cinnamoyl-CoA reductase; *CFAT*, coniferyl alcohol acetyltransferase; *CHD*, cinnamoyl-CoA hydratase-dehydrogenase; *CNL/AAE*, cinnamate:CoA ligase/acyl-activating enzyme; *COMT*, caffeic/5-hydroxyferulic acid O-methyltransferase; *CSE*, caffeoyl shikimate esterase; *EGS*, eugenol synthase; *HCT*, hydroxycinnamoyl transferase; *IGS*, isoeugenol synthase; *KAT*, 3-ketoacyl-CoA thiolase; *PAAS*, phenylacetaldehyde synthase; *PAL*, phenylalanine ammonialyase; *PAR*, phenylalanine reductase.

*Petunia* × *hybrida* cv ‘Mitchell Diploid (MD)’ is a well-characterized model plant to study flower volatiles, and many genes/transcripts/proteins involved in floral volatile benzenoid/phenylpropanoid (FVBP) biosynthesis have been characterized using this model (Fig. 1)^27–30^. Several steps downstream of 4CL have been identified and the functions of caffeoyl-CoA O-methyltransferase (CCoAOMT1)^31^, cinnamoyl-CoA reductase (CCR)^29,32^, cinnamyl alcohol dehydrogenase (CAD)^32^, coniferyl alcohol acyltransferase (CFAT)^33^, eugenol synthase1 (EGS1)^34^, and isoeugenol synthase1 (IGS1)^34^ have been reported for the biosynthesis of the phenylpropenes, eugenol and isoeugenol. Although several genes were characterized in other plant species, the biochemical steps from *p*-coumaric acid to caffeoyl-CoA have not yet been clearly investigated in Petunia.

The *p*-coumarate 3-hydroxylase (C3H) converts *p*-coumaroyl shikimate/quinate to caffeoyl shikimate/quinate^35^ and hydroxycinnamoyl transferase (HCT) participates in a reaction from *p*-coumaroyl CoA to caffeoyl CoA by exchanging CoA with shikimate or quinate as intermediates^36,37^. Recently, caffeoyl shikimate esterase (CSE) was also shown to be involved in phenylpropanoid biosynthesis. CSE converts caffeoyl shikimate into caffeic acid and then is combined with 4CL making caffeoyl-CoA, which can provide a bypass of the second reaction of HCT^38,39^.

Protein-protein associations are well known in these phenylpropanoid biosynthesis genes. The positive linear relations between PALs and C4Hs in Arabidopsis^40^, tobacco^11,41^, petunia^20^, and hybrid poplar^14^ have been established. Poplar C4H and C3H were reported to form heterodimeric and heterotrimeric membrane protein complexes^24^ and Arabidopsis C3H suppression is accompanied by reduced expression of *PAL1*, *C4H*, and *HCT*^42^. Bassard *et al*.^12^ showed co-localization and co-streaming of Arabidopsis C4H (CYP73A5) and C3H (CYP98A3) in the ER. The connecting HCT was also partially associated with the ER and C3H was important in driving the protein associations in Arabidopsis. Therefore, we hypothesized that the effects of *PhC3H* down regulation on the transcript accumulation of phenylpropanoid biosynthetic genes and that modification of *PhC3H* and/or *PhHCT* would alter the profile of petunia floral volatiles. To elucidate the role of the genes, we made knock-out constructs and transformed each construct into petunia. This research shows the reduction of *PhC3H* or *PhHCT* transcript resulted in change of the transcript level of FVBP genes and flower volatiles. Although additional research is required to illustrate *PhHCT* and *PhCSE* functions, we report the possibility of C3H as an important regulator of petunia MD FVBP biosynthesis.

## Results

### Identification of *C3H*, *HCT*, and *CSE* in Petunia

The cloned cDNA for *PhC3H* was 1790 base pairs (bp) in total length and included a 1533 bp open reading frame (ORF), which was predicted to encode for a protein composed of 511 residues with a predicted molecular weight of 58.3 kDa. The cloned cDNA for *PhHCT* was 1618 bp, contained a predicted 1305 bp ORF, which would result in 435 residues with a predicted molecular weight of 48.4 kDa. The cloned cDNA for* PhCSE* was 1149 bp in total length and included a predicted 933 bp ORF, which was predicted to encode for a protein composed of 311 residues with a predicted molecular weight of 35.0 kDa. The sequences for *PhC3H, PhHCT*, and *PhCSE* were submitted to NCBI under accession number KY679148, KY679147, and MF421742, respectively.

**Figure 2:**
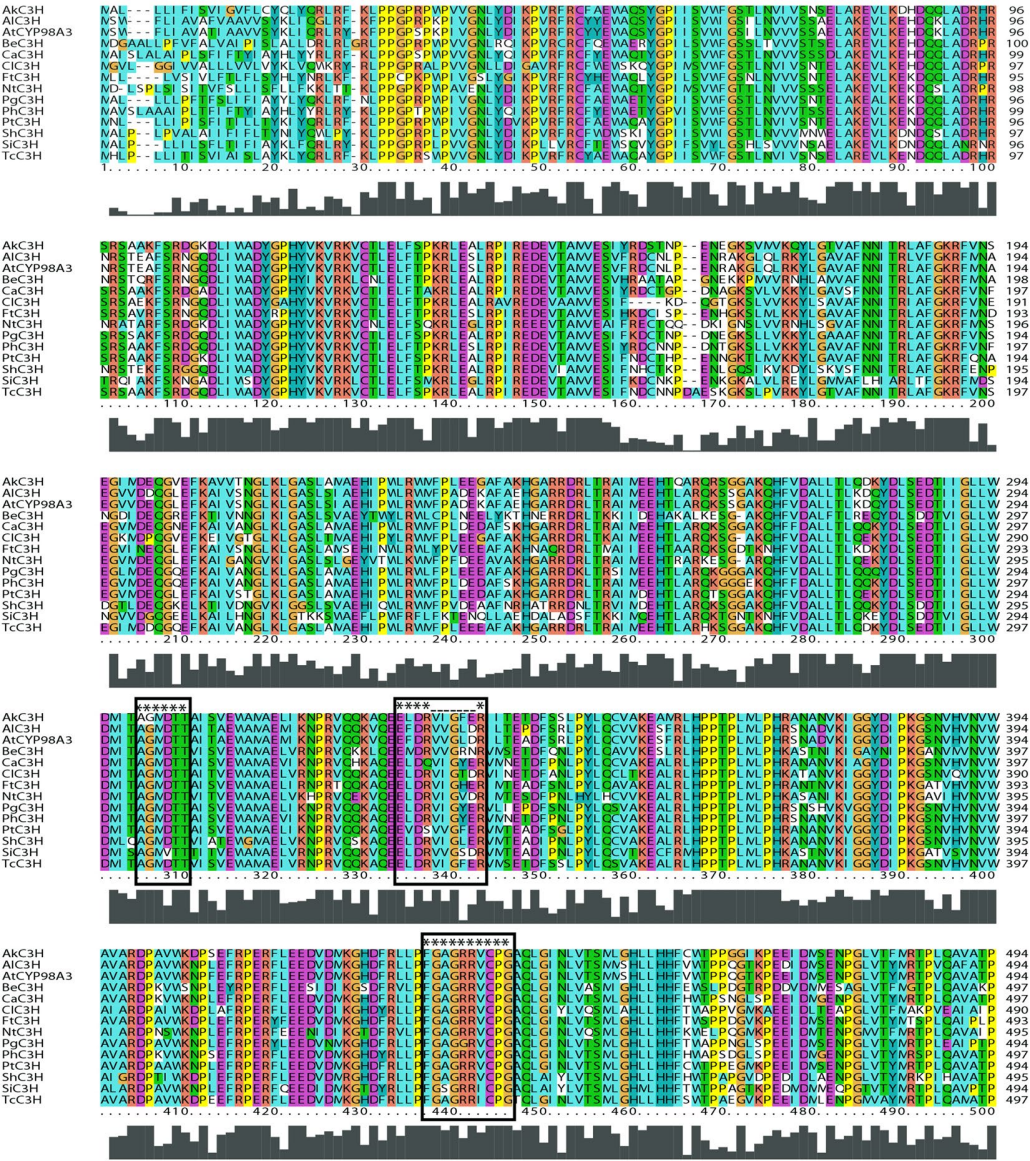
Predicted amino acid sequence alignment for C3H from various plants. The predicted amino acids of *PhC3H* contain all conserved domains of oxygen binding and activation (A/G-G-X-E/D-T-T/S), ERR triad (E-X-X-R…..R), and heme binding (F-X-X-G-X-R-X-C-X-G) for cytochrome P450 families. AkC3H, *Acacia koa*; AtCYP98A3, *Arabidopsis thaliana*; BeC3H, *Bambusa emeiensis*; CaC3H, *Capsicum annuum*; ClC3H, *Cunninghamia lanceolata*; FtC3H, *Fagopyrum tataricum*; NtC3H, *Narcissus tazetta*; PgC3H, *Platycodon grandifloras*; PhC3H, *Petunia* × *hybrida*; PtC3H, *Populus tomentosa*; ShC3H, *Sinopodophyllum hexandrum*; SiC3H, *Sesamum indicum*; TcC3H, *Theobroma cacao*.

The predicted amino acid sequence of *PhC3H* shared 94% homology to cytochrome P450 98A2-like protein from *Capsicum annuum* (NP_001311496), 85% identity to C3H from *Populus tomentosa* (AFZ78540), and 81% homology to CYP98A3 from *Arabidopsis thaliana* (OAP09214) (Fig. 2). PhC3H included all conserved domains of oxygen binding and activation (A/G-G-X-E/D-T-T/S), ERR triad (E-X-X-R…..R), and heme binding (F-X-X-G-X-R-X-C-X-G) for cytochrome P450 families^43^. PhC3H was predicted to localize to the endoplasmic reticulum (TargetP 1.1 Server) and had an N-terminal membrane anchoring peptide (TMHMM Server v.2.0). The predicted amino acid sequence of *PhHCT* shared 94% homology to HCT of *Nicotiana tabacum* (NP_001312552), 94% identity to shikimate O-HCT of *Capsicum annuum* (NP_001311756), and 78% homology to HCT of *Arabidopsis thaliana* (NP_199704) (see Supplementary Fig. S1). PhHCT contained a HHXXXDG and DFGWG motifs for BAHD superfamily and closed to hydroxycinnamoyl-CoA:shikimate/quinate hydroxycinnamoyl transferase (PLN02663) in a condensation superfamily^37,44,45^. The predicted subcellular localization for PhHCT was the cytoplasm (Plant-mPLoc). The predicted amino acid sequence of *PhCSE* shared 84% homology to predicted CSE of *Nicotiana tabacum* (XP_016470669), 82% identity to predicted CSE of *Solanum lycopersicum* (XP_004235722), and 75% homology to LysoPL2 of *Arabidopsis thaliana* (OAP14317) (see Supplementary Fig. S2). PhCSE belongs to α/β-hydrolase superfamily and contains nucleophile (GX-Nuc-XG)-acid-histidine catalytic triad, which is conserved motif among members of α/β-hydrolase family (see Supplementary Fig. S2)^46^. The predicted subcellular localization for PhCSE was in the cytoplasm (WoLF PSORT).

### Transcript accumulation of *PhC3H*, *PhHCT*, and *PhCSE* in MD

Since C3H, HCT, and CSE are involved in phenylpropanoid biosynthesis in other plant species, we hypothesized that relative transcript accumulation of *PhC3H*, *PhHCT*, and *PhCSE* would be highest in open flower and petal limb tissue, consistent with previously characterized FVBP genes^25,47–50^. Transcript accumulation was assayed using the ∆ΔCt qRT-PCR method with total RNA extracted from a spatial series of MD tissues: root, stem, stigma, anther, leaf, petal tube, petal limb, and sepal; along with total RNA from a staged floral developmental series of tissues including 11 consecutive stages MD flowers^48^.

**Figure 3:**
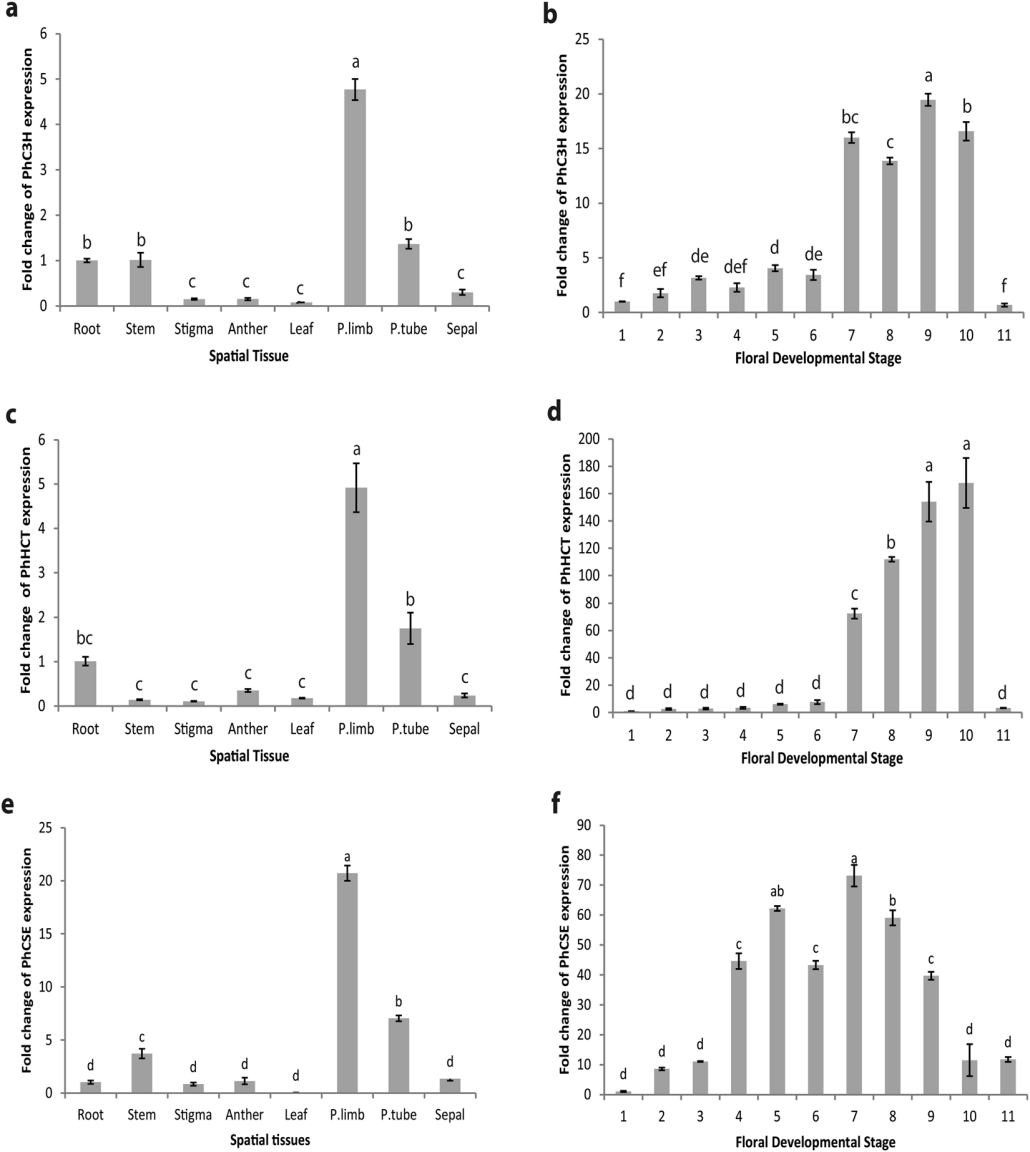
Transcript accumulation analyses of *PhC3H, PhHCT*, and *PhCSE* for spatial (**a**,**c**,**e**) and developmental stages (**b**,**d**,**f**). (q)RT-PCR was performed with ubiquitin as an internal reference. Error bar represents standard error and means separation was analyzed by Tukey test (one-way ANOVA, p < 0.05).

Spatial transcript accumulation was calculated based on that of root tissue where most transcripts of petunia volatile genes were lowest in previous studies^31,33,48,51^. The transcript accumulation of *PhC3H, PhHCT,* and  *PhCSE* was highest in petal limb tissue while lowest in reproductive organs, leaf and sepal tissues (Fig. 3a,c,e). An approximate five-fold increase of *PhC3H* and *PhHCT* transcript was detected in petal limb tissue compared to root tissue; whereas, an approximate twenty-fold increase of *PhCSE* transcript was detected for the same tissue comparison. The transcript accumulation level of *PhC3H* and *PhHCT* in root tissue relatively elevated compared to stigma, anther, leaf, and sepal tissue. These results do not consistent with the profiles of known FVBP genes of petunia^31,33,48,51^.

Floral developmental analyses demonstrated high levels of transcript accumulation only in open flowers from stage 7 to stage 10 for both *PhC3H* and *PhHCT* (Fig. 3b,d), while *PhCSE* transcript accumulation occurred earlier during flower development and showed a bell-shape with a peak at the flower opening (stage 7) (Fig. 3f). Focusing on flower developmental stage 9 (fully open corolla and sexually receptive flower), *PhC3H* transcript was accumulated approximately 20-fold more compared to stage 1 (initial flower bud). Similar trends were observed in the accumulation of *PhHCT* and *PhCSE* with an approximate 100-fold and 40-fold increase respectively. However, *PhCSE* accumulation peaked earlier, at flower stage 7 rather than stage 9, with approximately a 70-fold increase compared to stage 1.

### Transgenic *ir-PhC3H* and *ir-PhHCT* lines

**Figure 4:**
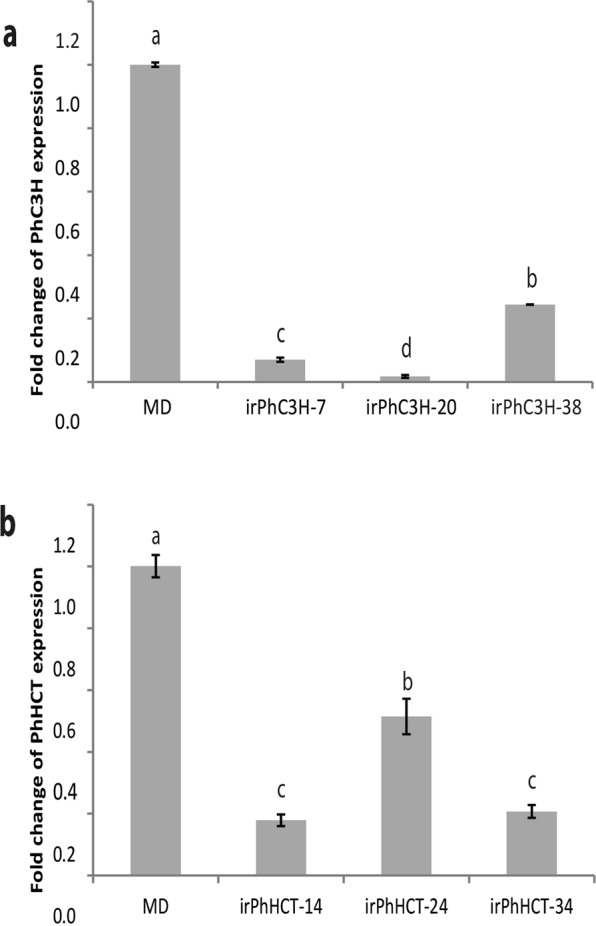
Transcript accumulation analyses in *ir-PhC3H* plants (**a**) and *ir-PhHCT* plants (**b**). (q)RT-PCR was performed with *PhFBP1* as an internal reference. Error bars represent standard error and means separation was analyzed by Tukey test (one-way ANOVA, p < 0.05).

Transgenic petunia lines were generated with reduced endogenous transcript for *PhC3H* and *PhHCT* using standard transformation and inverted repeat (ir) RNAi techniques driven by a constitutive promoter, pFMV^33,50–54^. Changes in overall plant growth, reproductive, or other morphological phenotypes were not observed. Transcript levels of endogenous *PhC3H* were decreased between 75.6 and 98.2% in flowers of representative, independent *ir-PhC3H* plants (*ir-PhC3H*-7, *ir-PhC3H*-20, and *ir-PhC3H*-38) compared to flowers from MD control plants (Fig. 4a). Endogenous *PhHCT* transcript levels were decreased in flower tissue of representative, independent *ir-PhHCT* plants (*ir-PhHCT-14*, *ir-PhHCT-24*, and *ir-PhHCT-34*) by approximately 48.7 to 82.3% compared to MD (Fig. 4b).

### Volatile analyses of *ir-PhC3H* and *ir-PhHCT* flowers

**Figure 5:**
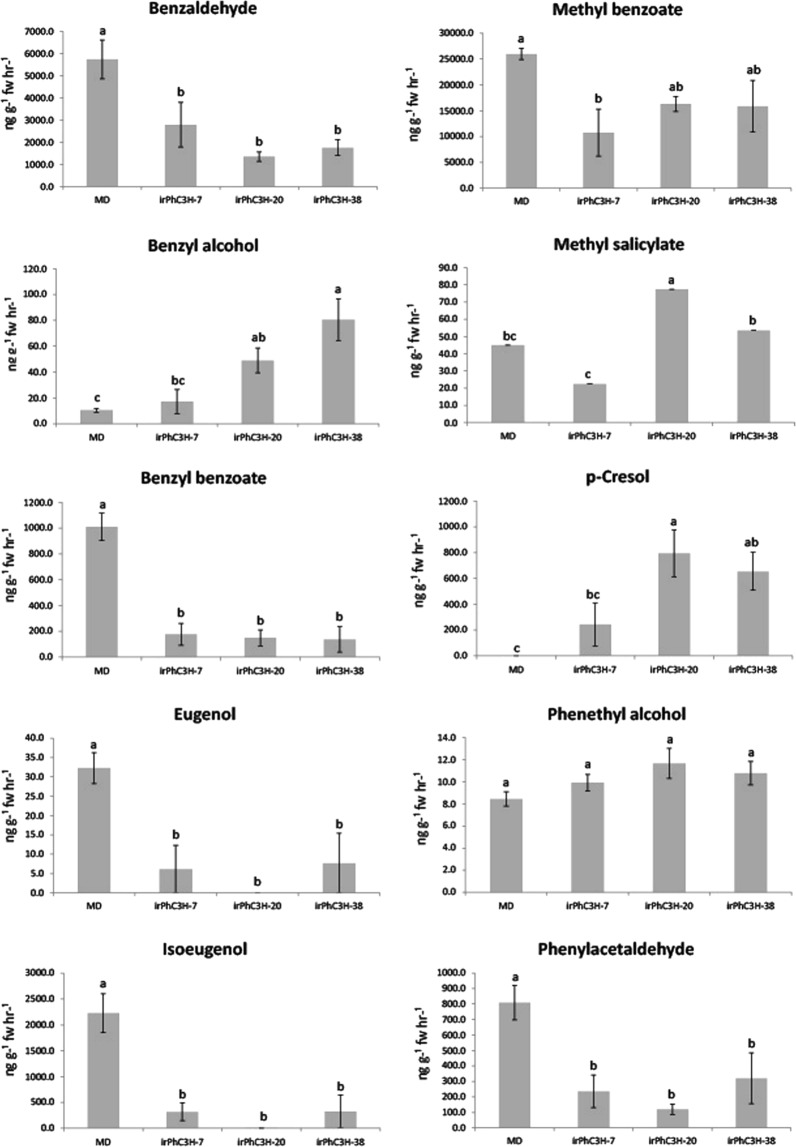
Effects of down-regulation of *PhC3H* on the floral volatiles. The open flowers (developmental stage 8) were used to collect volatiles at 18.0 h for 1 hr and the calculated emission was compared to MD plants. Error bars represnt standard error and means separation was analyzed by Duncan’s multiple range test (one-way ANOVA, p < 0.05).

Standard volatile collection and GC-MS analysis methods were used to compare floral volatile profiles of *ir-PhC3H* and *ir-PhHCT* lines to control MD plants. In general, *ir-PhC3H* lines showed reduced emission for five major petunia floral volatiles: eugenol, isoeugenol, benzyl benzoate, benzaldehyde, and phenylacetaldehyde. Compared to MD, *ir-PhHCT* lines were reduced in phenylpropene and benzyl benzoate emission and showed a trend toward increased emission of benzaldehyde and phenylacetaldehyde (Fig. 5, see Supplementary Fig. S3). The most abundant constituent of the MD floral volatile profile, methyl benzoate, was also reduced in *ir-PhC3H*-7 flowers, but to a lesser extent. Phenethyl alcohol was the only floral volatile not significantly different in all three *ir-PhC3H* lines compared to MD. The benzenoid *p*-cresol, which is a novel volatile molecule for the MD genetic background, was detected from flowers of *ir-PhC3H* lines (Fig. 5).

Floral volatile emission from *ir-PhHCT* lines were significantly reduced for the phenylpropene volatiles, eugenol and isoeugenol, and the two conjugated volatiles benzyl benzoate and phenethyl benzoate (see Supplementary Fig. S3). The *ir-PhHCT* lines exhibited elevated emission of benzaldehyde, benzyl alcohol, and phenylacetaldehyde compared to MD. Methyl benzoate was also increased in *ir-PhHCT*-14 flowers, but to a lesser extent. *P*-cresol was not detected from any *ir-PhHCT* flowers (data not shown).

### Transcript accumulation of FVBP related genes in *ir-PhC3H* and *ir-PhHCT* flowers

**Figure 6:**
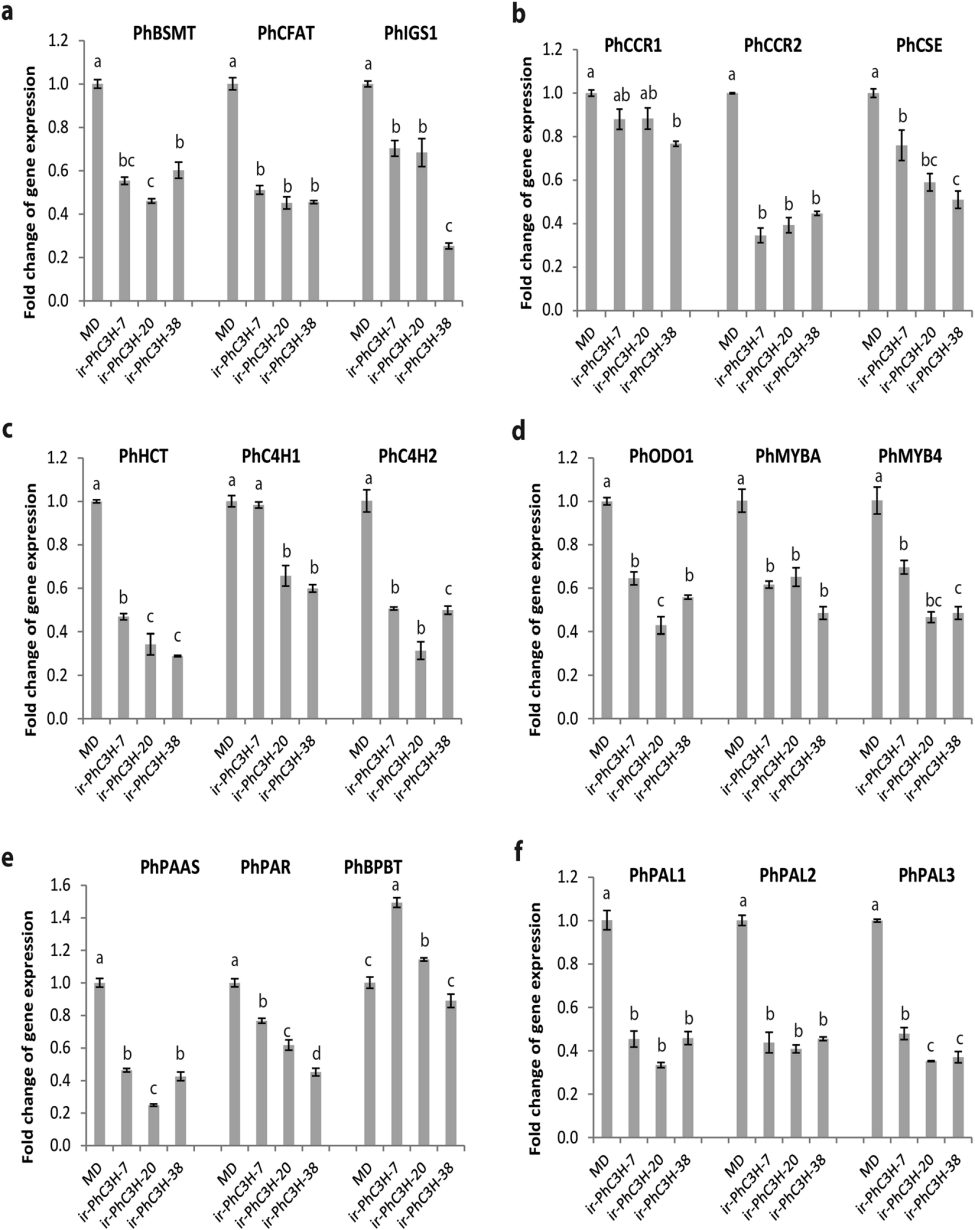
Transcript accumulation analyses of phenylpropanoid biosynthesis genes in *ir-PhC3H* plants. (q)RT-PCR was done with *PhFBP1* as an internal reference. Error bars represnent standard error and means separation was analyzed by Tukey test (one-way ANOVA, p < 0.05).

C3H is reported as a key protein concerning regulation of metabolically related protein aggregates^12^. As PhC3H transcript levels were low in petunia flowers, most of the volatiles were reduced (Fig. 5). Transcript accumulation of the metabolically related genes, *PhC4Hs*, *PhPALs*, and *PhHCT* showed decreased levels in *ir-PhC3H* flowers compared to MD. Many other FVBP genes including *PhBSMT*, *PhCCR2*, *PhCFAT*, *PhCSE*, *PhIGS1*, *PhMYBA*, *PhMYB4*, *PhODO1*, *PhPAAS*, and *PhPAR* demonstrated reduced transcript accumulation in *ir-PhC3H* flowers (Fig. 6). However, down regulation of *PhHCT* did not affect transcript accumulation of *PhCSE* as much as that of *PhC3H* (see Supplementary Fig. S4).

## Discussion

The transcript accumulation profile, enzyme function, protein localization, and protein-protein interactions of coumarate 3-hydroxylase (C3H) have been investigated in model plant systems like Arabidopsis^12,35,42,43,55,56^ and poplar^24,57^. C3H is an integral protein feature of the cellular machinery leading to produce monolignols which serve as precursors to lignin and lignin production in plants^58^. Coniferyl alcohol, a common monolignol, can serve as a precursor to volatile phenylpropene biosynthesis in petunia floral tissue where the requirement for lignin is relatively low^59^. Around 7:00 PM, a petunia flower can emit over 10 ug*gFW^−1^*h^−1^ of a volatile phenylpropene, isoeugenol^48,50^, and the rate of phenylpropene and benzenoid emission is least influenced by a limiting pool of phenylalanine, the initial substrate for the phenylpropanoid pathway, indicating a strong regulation of carbon flux toward monolignol production^52,60^. When arogenate dehydratase, which converts arogenate to phenylalanine, was down regulated in petunia, the emission of many petunia volatiles was reduced^60^.

Throughout this work, three transcripts were cloned and sequenced from *Petunia* × *hybrida* cv ‘MD’ flower tissue. Single and unique transcripts with homology to C3H, HCT, and CSE were identified using publicly available genomic and transcriptomic databases, although the possibilities for the multiple copies of each gene family member could not be excluded (Fig. 2, see Supplementary Figs S1 and S2). All three petunia transcripts accumulated to their relatively highest levels in flower limb tissue compared to other parts of the plant (Fig. 3), which is in line with FVBP network transcripts^48^. *PhC3H* and *PhHCT* exhibited typical accumulation patterns of genes involved in FVBP biosynthesis during floral development^20,25,29,31,33,48,49,51–53,60^. The transcript levels were relatively low in young or developing floral buds, highest in open flowers, and then showed a dramatic reduction at senescence (Fig. 3)^48^. Compared to *PhC3H*, *PhHCT* and other known genes in the FVBP biosynthesis pathway, *PhCSE* transcript accumulated earlier during development and displayed an almost normal distribution rather than a developmentally-delayed distribution (Fig. 3). Interestingly, *PhCSE* followed a developmental bell-shaped accumulation profile similar to that of the petunia R2R3-MYB transcription factor, PhEOBII, which appears to have a positive regulatory effect on flower opening and FVBP biosynthesis^61,62^.

Petunia RNAi (*ir*) lines for *PhC3H* and *PhHCT* were generated to test the effects of reduced transcript levels of each gene on FVBP pathway. Multiple lines of *ir-PhC3H* showed a reduction of endogenous *PhC3H* transcript by 75.6–98.2% in floral tissue, and *ir-PhHCT* lines were reduced in endogenous transcript by 48.7–82.3% (Fig. 4). It is unclear whether a reduction of *PhHCT* transcript beyond 82.3% is lethal, but no obvious growth phenotypes were observed any of the *ir-PhHCT* or *ir-PhC3H* lines. Floral volatile analysis of the *ir-PhC3H* lines demonstrated a clear but unexpected volatile phenotype compared to the control petunia volatile emission phenotype (Fig. 5). The reduction of endogenous *PhC3H* resulted in very low levels of emitted phenylpropenes, isoeugenol and eugenol, as expected. Additionally, most of the emitted benzenoid volatiles were also reduced, along with phenylacetaldehyde which is generated directly from the initial substrate of the phenylpropanoid pathway, phenylalanine. A novel floral volatile molecule, *p*-cresol was detected from *ir-PhC3H* lines at considerable concentrations (~200–800 ng*gFW^-1^*h^−1^). It was an unexpected product because *p*-cresol has not been reported in FVBP biosynthesis of MD before but detected in other floral volatile profiles such as *Petunia* × *hybrida* (V26) having purple flowers, *Satyrium pumilum* (African orchid), and *Jasminum polyanthum* (Pink Jasmin)^63–65^. *P*-cresol is known to be converted from 4-hydroxyphenylacetic acid which is derived from tyrosine^65,66^.

The volatile phenotype of the *ir-PhC3H* lines suggested that a disruption of C3H activity cause a downregulation of the majority of the phenylpropanoid pathway in petal tissue of petunia flowers. Based on the transcript accumulation assay, 16 of the 18 FVBP related genes demonstrated clear reductions in the *ir-PhC3H* lines compared to controls (Figs 1, 6). Known positive and negative regulating transcription factors^20,61,66^, core phenylpropanoid pathway enzymes^20,25^, enzymes that produce specific FVBP compounds^33,67^, and even enzymes responsible for biochemical steps after C3H^29,31^ were all significantly reduced in transcript accumulation. For example, the small family of phenylalanine ammonia-lyase (PAL1, 2, 3) transcripts was reduced 52.3–66.8%, which would severely limit phenylpropanoid metabolism in petal tissue, especially at the elevated rates normally found in petunia flower petal tissue.

It is unclear at this point what mechanisms are involved in the downregulation of the general phenylpropanoid pathway. In Arabidopsis and poplar, C3H appears to be a major driver of protein-protein interaction at the ER, where C4H and C3H form homodimer and heterodimer protein complexes with elevated enzymatic activity^12,24,37,43^. The P450 protein complexes can associate with soluble phenylpropanoid pathway enzymes like PALs, 4CLs, and HCT to form supramolecular structures. These large, ER tethered protein aggregates are thought to concentrate required enzymes to accommodate for a high metabolic demand of the phenylpropanoid pathway in specific conditions or tissues^12^. We demonstrated that the down regulation of *PhC3H* in petunia resulted in the change of flower volatiles and expression of related FVBP genes, but further studies are required to elucidate the role of PhC3H in the stability of a large phenylpropanoid related protein complex in petunia flower limb tissue.

## Materials and Methods

### Plant materials and Cloning

*Petunia* × *hybrida* cv ‘MD’ was used as a control and genetic background for all experiments. Plants were grown in glass greenhouses as previously described by Dexter *et al*.^33^.

Multiple data sources including the National Center for Biotechnology Information (NCBI - http://www.ncbi.nlm.nih.gov), the Sol Genomics Network (SGN - https://solgenomics.net), and the 454 petunia database (http://biosrv.cab.unina.it/454petuniadb/protocol.php) were employed to search for petunia nucleotide sequences with similarity to *Arabidopsis thaliana p-coumarate 3-hydroxylase* (*AtC3H*, AT2G40890), *Nicotiana tabacum hydroxycinnamoyl transferase* (*NtHCT*, AJ507825), and *Arabidopsis thaliana caffeoyl shikimate esterase* (*AtCSE*, AT1G52760). The target petunia sequences were collected and assembled into contigs using a software package (Vector NTI Advance™ 11.3) and a SMARTer™ RACE cDNA Amplification Kit (Clontech Laboratories, Inc., Mountain View, CA) according to the manufacturer’s protocol. This approach resulted in three *in silico* candidate sequences for *PhC3H*, *PhHCT*, and *PhCSE*. The full sequences were amplified using PfuTurbo DNA Polymerase (Agilent Technologies, Santa Clara, CA) (primers on Table S1) and cloned into a pGEM-T easy vector (Promega, Madison, WI) using similar methods as Colquhoun *et al*.^20^. Nucleotide sequencing with multiple clones from multiple amplifications was performed at an on campus Sanger sequencing core (Interdisciplinary Center for Biotechnology Research, University of Florida, FL) using Big Dye V1–2. The resulted high-quality sequence was then used as a query to search the petunia genome database at SGN (https://solgenomics.net), which supported that each sequence most likely originating from a single locus.

### Transgenic *PhC3H* RNAi (*ir-PhC3H*) and *PhHCT* RNAi (*ir-PhHCT*) plants

A 302 bp sequence of *PhC3H* and a 335 bp sequence of *PhHCT* were amplified for RNAi vector construction (primers on Table S1). The RNAi gene driven by a flower specific constitutive promoter, pFMV in pHK vector was introduced into MD leaf discs using *Agrobacterium*-mediated transformation methods^68^. Detailed methods for this procedure have been described by Dexter *et al*.^33^ and Underwood *et al*.^50^. All T_0_ plant tissues were collected for floral volatile analyses and transcript accumulation analyses, and then the flowers were self-pollinated.

### Analyses of transcript accumulation

To observe transcript accumulation based on spatial and flower development, petunia MD tissues were collected following the method of Colquhoun *et al*.^48^. The spatial series consisted of root, stem, stigma, anther, leaf, petal tube, petal limb, and sepal. The developmental series included 11 stages of flowers, bud < 0.5 cm (stage 1); bud 0.5 to 1.5 cm (stage 2); bud 1.5 to 3.0 cm (stage 3); bud 3.0 to 5.0 cm (stage 4); bud fully elongated, 5.0 to 6.5 cm (stage 5); flower opening 0 to 2 cm limb diameter (stage 6); flower fully open days 0 (stage 7), 1 (stage 8), 2 (stage 9), and 3 (stage 10); senescing flower (flower open day 7 for MD) (stage 11). All tissues were collected in liquid N_2_ at 16.00 h and stored at -80 °C with two biological replications.

Total RNA was extracted as previously described^67^ using TriZOL^TM^ (ThermoFisher Scientific, Waltham, MA), treated with TURBO^TM^ DNA-free^TM^ (Ambion Inc., Austin, TX), and then purified using the RNeasy® Mini protocol (Qiagen Co., Valencia, CA). 50 ng µL^−1^ of RNA was prepared after measuring the concentration using a NanoDrop^TM^ 2000c spectrophotometer (ThermoFisher Scientific, Waltham, MA). Transcript accumulation was analyzed with semi-quantitative (sq)RT-PCR using a One-step RT-PCR kit (Qiagen Co., Valencia, CA) and with ∆ΔCt quantitative (q)RT-PCR using Power SYBR® Green RNA-to-CT^TM^ 1-Step kit and StepOnePlus^TM^ real-time PCR system (ThermoFisher Scientific, Waltham, MA). Based on the nucleotide arrangement of full sequences, primers were designed using Primer3 (http://biotools.umassmed.edu/bioapps/primer3_www.cgi) (primers on Table S1). To analyze transcript accumulation of other FVBP genes in *ir-PhC3H* plants, ∆ΔCt quantitative (q)RT-PCR was performed with petunia benzoyl-CoA:benzylalcohol/2-phenylethanol benzoyltransferase (*PhBPBT*; AY611496), benzoic acid/salicylic acid carboxyl methyltransferase (*PhBSMT*, AY233465), cinnamoyl-CoA reductases (*PhCCR1*, KF040494 and *PhCCR2*), *PhC4H1* (HM447144), *PhC4H2* (HM447145), *PhCFAT* (DQ767969), *PhCSE* (AT1G52760), *PhHCT* (KY679147), *PhIGS1* (DQ372813), *PhMYBA* (EU374207), *PhMYB4* (HM447143), ODORANT1 (*PhODO1*, AY705977), phenylacetaldehyde synthase (*PhPAAS*, DQ243784), PhPALs (*PhPAL1*, AY705976; *PhPAL2*, CO805160; *PhPAL3*), and phenylalanine reductase (*PhPAR*) genes. ∆ΔCt (q)RT-PCR of *PhCSE* was also performed in *ir-PhHCT* plants to test an effect of reduced *PhHCT* transcript on other related FVBP biosynthesis genes. The *PhFBP1* (M91190) or *PhUbiq* (SGN-U207515) was used as an internal standard to compare expression of each gene. All ∆ΔCt (q)RT-PCR data was analyzed using 2^−∆ΔCt^ method^69^, while nonparametric statistical analyses were conducted using Kruskal-Wallis test with the JMP Pro v.12 statistical software package (SAS Institute Inc., Cary, NC). P-values were computed at significance level (alpha = 0.05).

### Volatile collection

Petunia flowers were harvested at 16.00 h and volatiles were collected for 1 hour in glass tubes using a push-pull dynamic headspace collection system as previously described^33,51,70^. Volatiles collected from at least three biological replicate flowers on glass columns containing approximately 50 mg HaySep Q 80–100 porous polymer adsorbent (Hayes Separations Inc., Bandera, TX) were eluted with methylene chloride. Quantification of volatiles from the elution matrix was performed on an Agilent 7890A Series gas chromatograph (GC) equipped with an Agilent 5977A single quadrupole mass spectrum detector (MSD). Parameters of the GC were used as follows: Helium carrier gas fixed at 11.5 psi, split injector at 20:1 split, inlet temperature 220 °C, injection volume 2 uL, and the syringe wash solvents were acetone and hexane. Sample analytes were separated using an equipped DB-5 column (Agilent Technologies, Santa Clara, CA, USA). Oven temperatures were programmed as follows: the initial oven temperature of 40 °C was held for 0.5 minutes then ramped 5 °C*minute^−1^ to 250 °C and held for 4 minutes. The MSD was equipped with an extractor ion source and tuned for sensitivity and mass accuracy just prior to sample analysis. Parameters for the MSD were maintained as follows: MSD transfer line temperature 280 °C, MS source temperature 230 °C, MS quad temperature 150 °C, solvent delay of 4.40 minutes, mass scan range 40–205 m/z with a threshold of 150. Data was acquired using Agilent MassHunter Workstation Acquisition (version, Agilent Technologies, Santa Clara, CA) and processed using Agilent’s MassHunter Quantitative Analysis program. Compound identity was verified by extracting and comparing the mass spectral data of each compound peak to the 2011 NIST mass spectral library and comparing retention time and mass spectral profiles to authentic standards run under identical machine parameters. Volatile mass emission rates (ng*gFW^−1^*hr^−1^) were calculated based on each compound individual peak area relative to the peak area of an elution standard, nonyl acetate, within each sample and standardized for each sample corresponding biological mass. Dilutions for volatile standards were run on the GC-MS in duplicate to obtain a response factor for each compound that was used in the calculation of volatile emission mass. Mean separation and comparison of *ir-PhC3H* and *ir-PhHCT* floral volatiles to MD controls were performed using Duncan’s multiple range test (one-way ANOVA, P < 0.05) with the JMP Pro v.12 statistical software package (SAS Institute Inc., Cary, NC).

## Supplementary information

Supplementary figures

## Data Availability

All submitted manuscripts including figures and tables are available on Scientific report.
