# Supplementary material for: Down regulation of p-coumarate 3-hydroxylase in petunia uniquely alters the profile of emitted floral volatiles
Source: Sci Rep. 2019 Jun 20;9:8852. doi: 10.1038/s41598-019-45183-2 (PMC6586934; doi:10.1038/s41598-019-45183-2)
Supplement: Supplementary file 1 — Supplementary figures [file 41598_2019_45183_MOESM1_ESM.pdf]

TITLE PAGE

Down regulation of *p-coumarate 3-hydroxylase* in petunia uniquely alters the profile of emitted floral volatiles

Joo Young Kim[1], Robert T. Swanson[1], Maria I. Alvarez[1], Timothy S. Johnson[1], Keun H. Cho[1], David G. Clark[1], and Thomas A. Colquhoun[1]*

[1]Environmental Horticulture Department, Plant Innovation Center, Institute of Food and Agricultural Sciences, University of Florida, Gainesville, FL 32611, USA

*Corresponding author: Thomas A. Colquhoun, University of Florida, 1523 Fifield Hall, PO Box 110670, Gainesville, FL 32611, USA, Office: 352-273-4584, Fax: 352-392-3870, email: ucntcme1@ufl.edu

Authors' Email:
Joo Young Kim, jkim@ufl.edu
Robert T. Swanson, rswanson5335@ufl.edu
Maria I. Alvarez, ialvarez@ehs.ufl.edu
Timothy S. Johnson, timsjohnson@ufl.edu
Keun H. Cho, kencho@ufl.edu
David G. Clark, geranium@ufl.edu
Thomas A. Colquhoun, ucntcme1@ufl.edu

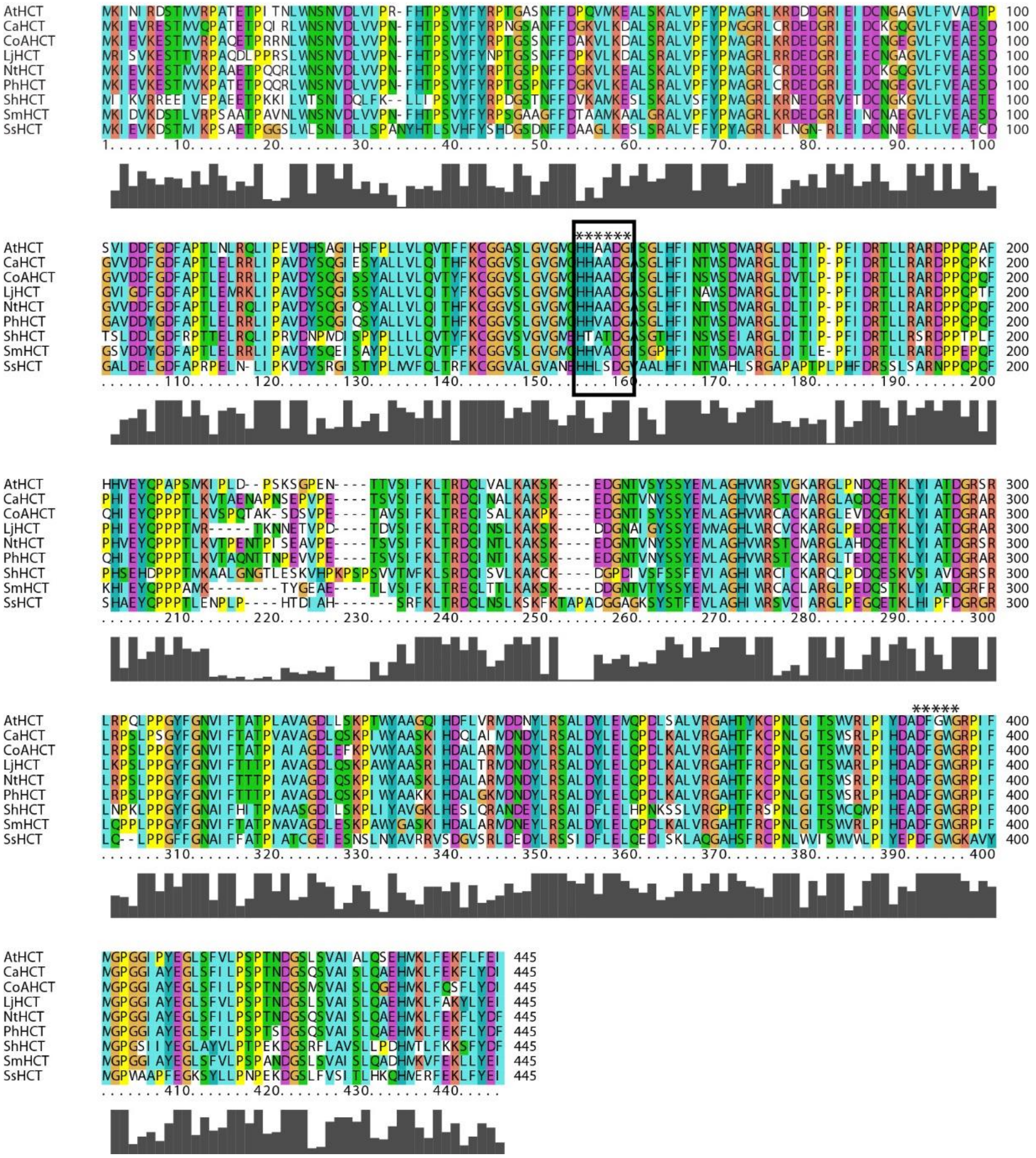

Figure S1. Predicted amino acid sequence alignment for HCT from various plants. The multiple alignment (ClustalX 2.0) was used to align the sequences. The predicted amino acids of *PhHCT* contain HHXXXDG and DFGWG motifs for BAHD family.

AtHCT, *Arabidopsis thaliana* HCT(NP_199704); CaHCT, *Capsicum annuum* HCT (NP_001311756); CoAHCT, *Coffea arabica* HCT (CAJ40778); LjHCT, *Lonicera japonica* HCT (AFS68800); NtHCT, *Nicotiana tabacum* HCT (NP_001312552); PhHCT, *Petunia* x *hybrida* HCT; ShHCT, *Sinopodophyllum hexandrum* HCT (ADB44897); SmHCT, *Salvia miltiorrhiza* HCT (ADG46003); SsHCT, *Solenostemon scutellarioides* HCT (CAK55166).

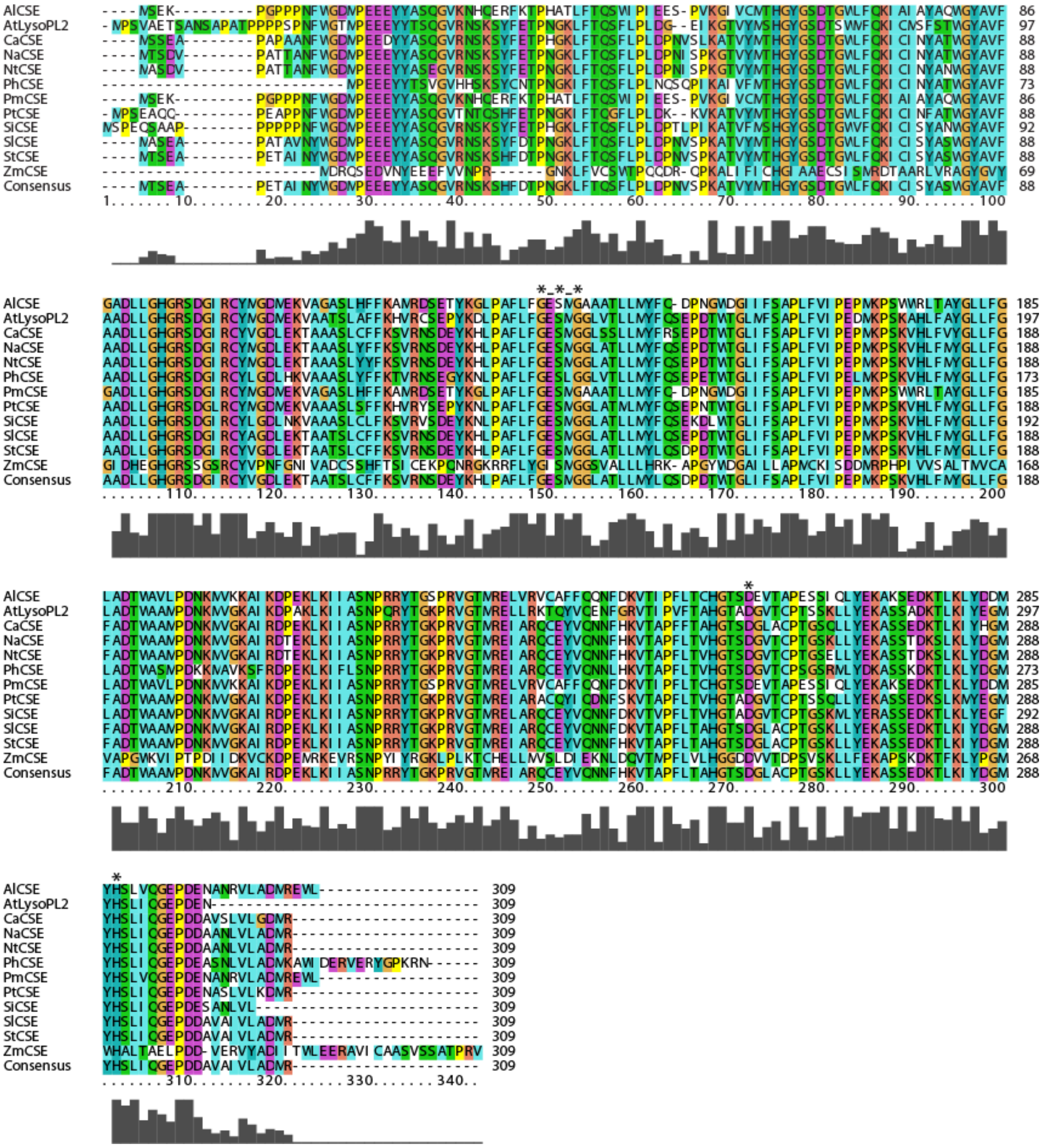

Figure S2. Predicted amino acid sequence alignment CSE from various plants. The multiple alignment (ClustalX 2.0) was used to align the sequences. The predicted amino acids of *PhCSE* contain nucleophile (GX-Nuc-XG)-acid-histidine catalytic triad which is conserved motif among members of α/β-hydrolase family.

AlCSE, *Arabidopsis lyrata subsp. lyrata* CSE (XP_020891585); AtLysoPL2, *Arabidopsis thaliana* LysoPL2 (OAP14317); CaCSE, *Capsicum annuum* CSE (XP_016562751); NaCSE, *Nicotiana attenuata* CSE (XP_019241751); NtCSE, *Nicotiana tabacum* CSE (XP_016470669); PmCSE, *Pinus massoniana* CSE (AKD43981); PtCSE, *Populus tomentosa* CSE (ALM55679); SlCSE, *Solanum lycopersicum* CSE (XP_004235722); StCSE, *Solanum tuberosum* CSE (XP_006341666); SiCSE, *Sesamum indicum* CSE (XP_011072262); ZmCSE, *Zea mays* CSE (XP_008674851).

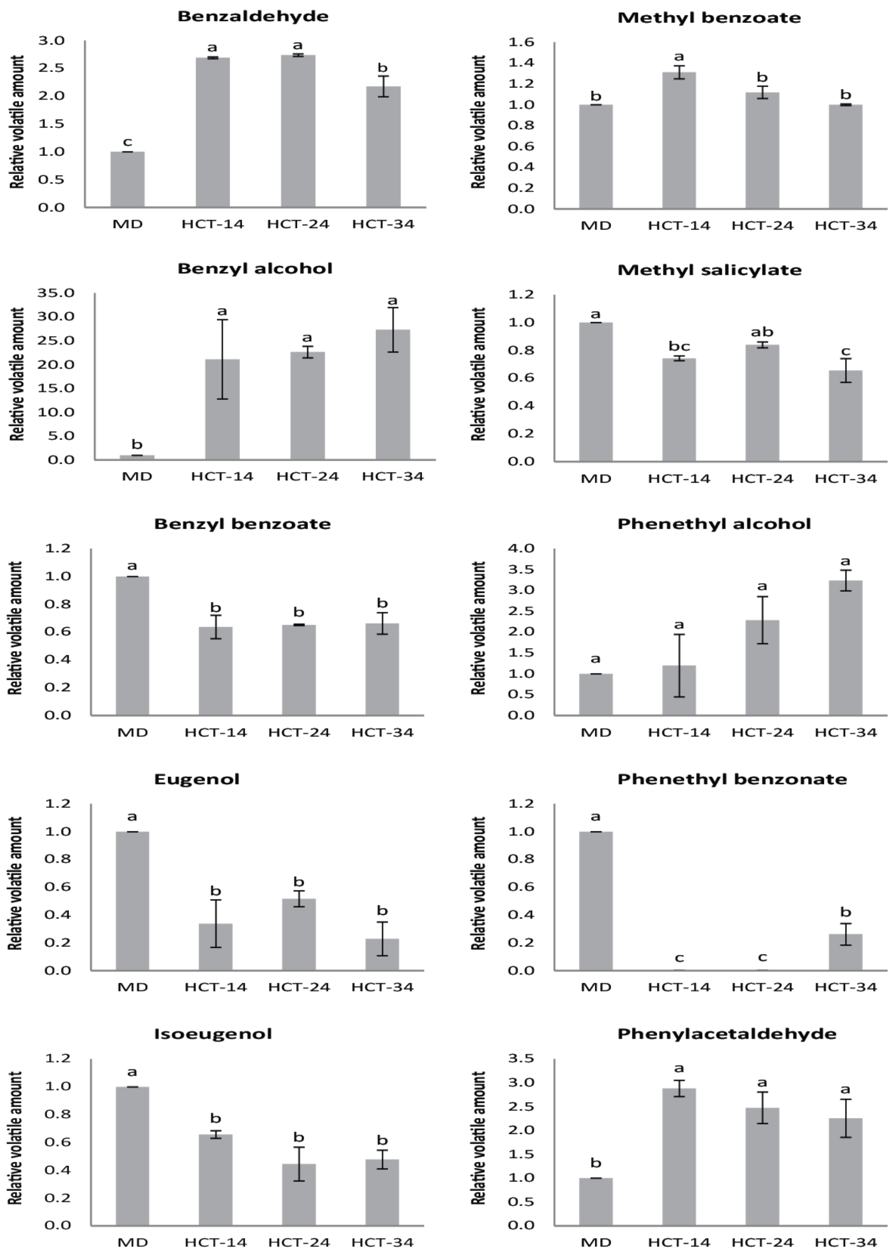

Figure S3. Effects of down-regulation of *PhHCT* on petunia floral volatiles. The open flowers (developmental stage 8) were used to collect volatiles at 18.0 h for 1hr and the calculated emission was compared to MD plants. Error bars represent standard error and means separation was analyzed by Duncan's multiple range test (one-way ANOVA, $p<0.05$).

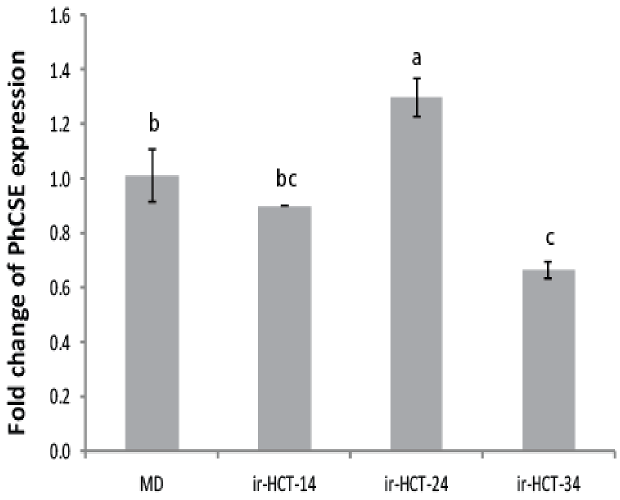

Figure S4. Transcript accumulation analysis of *phCSE* gene in *ir-PhHCT* plants. The open flowers (developmental stage 8) were used and relative quantities of the transcript of *PhCSE* gene were compared to MD plants. (q)RT-PCR was done with three biological replicates with *PhFBP1* as an internal reference. Error bars represnent standard error and means separation was analyzed by Tukey test (one-way ANOVA, $p < 0.05$).

Table S1. Primer sequences used in this research.

Name	Forward	Reverse
PhC3H-full	5'-ACATGGGGAACCCAAAAA-3’	5'-TGTTTGCTCCTGGGTAATCC-3’
PhHCT-full	5’-AGCTGTTGGAAAATCAAGAATC-3’	5’-GGTTTCTTTCAAAAGTCATACAAGA-3’
PhCSE-full	5’-GGACCTAACTTCTGGGGTGAC-3’	5’-ATGCCAAAGACCAAGATGATCA-3’
PhC3H-RNAi	5’-TTACCCGAGCCATTATGGAG-3’	5’-GTATGGGAGGTTGGGGAAGT-3’
		5’-AGGTAACAAGCCCAGGGTTC-3’
PhHCT-RNAi	5’-GGAGCTTCAGGTCTTCACTT-3’	5’-TTGCAAGTGGATCGCCAT-3’
		5’-TTAAGCCTTCATAAGCAATA-3’
PhBPBT-qPCR	5'-AGTGATGCACCCGGTCTTGT-3'	5'-CAATTCTCGGCACCACACTG-3'
PhBSMT-qPCR	5'-TTTTCAGTGGAGTGCCTGGTT-3'	5'-GGCACCTGAGATAGCCACATG-3'
PhC3H-qPCR	5’-CGTCTCCGTTACAAGCTTCC-3’	5’-AGCAGCCGATCTACTCCTATG-3’
PhC4H1-qPCR	5’-AGCAGGTGTAACAAACTGCAA-3’	5’-AAACTGGGACAGGGATAGGA-3’
PhC4H2-qPCR	5’-AACTTGTCCAAACAAAAATGGA-3’	5’-TGGCAATTTAAAACGTTTGCT-3’
PhCCR1-qPCR	5’-CTGAGGCATCTGGTCGGTAT-3’	5’-TGGTTTTACCCTTGGCTTG-3’
PhCCR2-qPCR	5’-TCCACACAGCTTCACCTGTTAC-3’	5’-CACATCGTTGGAAAGAATTGG-3’
PhCFAT-qPCR	5'-AGGCAACTCGCAATGGAAGT-3'	5'-AGGCGCTGAAACACTCCAAT-3'
PhCSE- qPCR	5'-ATTTGGGGAATCACTTGGTG-3'	5'-CCTTGGATGGCTTCATTAGC-3'
PhFBP1-qPCR	5’-TGCGCCAACTTGAGATAGCA-3’	5’-TGCTGAAACACTTCGCCAATT-3’
PhHCT-qPCR	5'-GACTATGGTGAAGCCAGCAA-3'	5'-ACATAGCCTCCCAGCCATAG-3'
PhIGS1-qPCR	5'-AGGAAGATGTCGCAGCCTACA-3'	5'-GTTTGCTGCCCTTGGATCAT-3'
PhMYB4-qPCR	5'-AACAATTTCTTTTGCTGCTGGAA-3'	5-TTCATCGTCCTTGATTTGTTCAA-3'
PhMYBA-qPCR	5'-CCCCATATATGTGAGTTAAGTGG-3'	5'-CCATAGGCACCTCCATGCAT-3'
PhODO1-qPCR	5'-TGCTTCAACCATGTCGAATTG-3'	5'-TCCGTGCCTGTTCTCTACGTT-3'
PhPAAS-qPCR	5'-CCAACCCGAACCAATTGAGA-3'	5'-CCTGGGAAAATATCGCTTCGA-3'
PhPAL1-qPCR	5’-GCTAGGCGGTGAGACGCTAA-3’	5’-CTCGGACAGCTGCACTGTCA-3’
PhPAL2-qPCR	5’-ACTGGCAGGCCTAATTCCAA-3’	5’-GCGAAACGCTTCTTCAGCAT-3'
PhPAL3-qPCR	5’-ATGGCGAAAATGAGAAGAATG-3’	5’-GGCCACTAACTCATCCTCAAA-3’
PhPAR-qPCR	5'-AAGCCATTTCCGCCAAAGTACC-3'	5'-GTACAGAAGCACGTTCACAA-3'
PhUbiq-qPCR	5’-GTCGATGGTCTTTGTTAGTGTTGTGT-3’	5’-CAGAAACAGGAGCCAATTAAAGCACT-3’
